# Supra-personal cognitive control and metacognition

**DOI:** 10.1016/j.tics.2014.01.006

**Published:** 2014-04

**Authors:** Nicholas Shea, Annika Boldt, Dan Bang, Nick Yeung, Cecilia Heyes, Chris D. Frith

**Affiliations:** 1 Department of Philosophy, King's College London, Strand, London WC2R 2LS, UK; 2 Department of Experimental Psychology, University of Oxford, South Parks Road, Oxford, OX1 3UD, UK; 3 Calleva Research Centre for Evolution and Human Sciences, Magdalen College, High Street, Oxford OX1 4AU, UK; 4 Interacting Minds Centre, Aarhus University, Jens Chr. Skous Vej 4, Building 1483, 8000 Aarhus, Denmark; 5 All Souls College, High Street, Oxford OX1 4AL, UK; 6 Wellcome Trust Centre for NeuroImaging at UCL, 12 Queen Square, London WC1N 3BG, UK

## Abstract

The human mind is extraordinary in its ability not merely to respond to events as they unfold but also to adapt its own operation in pursuit of its agenda. This ‘cognitive control’ can be achieved through simple interactions among sensorimotor processes, and through interactions in which one sensorimotor process represents a property of another in an implicit, unconscious way. So why does the human mind also represent properties of cognitive processes in an explicit way, enabling us to think and say ‘I’m sure’ or ‘I’m doubtful’? We suggest that ‘system 2 metacognition’ is for supra-personal cognitive control. It allows metacognitive information to be broadcast, and thereby to coordinate the sensorimotor systems of two or more agents involved in a shared task.

## Highlights

• We propose a ‘dual systems’ framework for thinking about metacognition.
• System 1 metacognition is for ‘intra-personal’ cognitive control.
• System 2 metacognition is for ‘supra-personal’ cognitive control.
• The latter allows agents to share metacognitive representations.
• This sharing creates benefits for the group and facilitates cumulative culture.

## A novel framework for metacognition research

Converging theoretical and empirical research suggests that most animals implicitly represent properties of their cognitive processes and use these for cognitive control (see Glossary 1, 2, 3) 4, 5, 6. This challenges the view that only humans have metacognition [7], which is broadly defined as ‘cognition about cognition’, and raises the question why humans, unlike other animals, not only implicitly but also explicitly represent properties of their cognitive processes.

In this opinion article, we propose a ‘dual systems’ framework for thinking about metacognition. In our framework, metacognition is composed of a cognitively ‘lean’ system, system 1 metacognition, which operates implicitly and is for the control of processes within one agent (intra-personal cognitive control), and a cognitively ‘rich’ system, system 2 metacognition, which is likely to be unique to humans and is for the control of processes within multiple agents (supra-personal cognitive control). Whereas the former system is found in many animals, the latter system is likely to be unique to humans. Our ‘lean’ account of metacognition clarifies the minimal requirements for metacognition and thereby offers a conceptual anchor in a literature where there are many conflicting assumptions about what counts as metacognition. Further, our ‘rich’ account of metacognition addresses why humans evolved the ability to explicitly represent properties of their cognitive processes: by sharing and discussing these representations, agents can engage in novel forms of adaptive group behaviour and build cumulative culture.

### Metacognition and its relation to cognitive control

The broad definition of metacognition as ‘cognition about cognition’ is often interpreted widely ([8], p. 170), to include any cognitive process that receives information from and has a controlling influence on another cognitive process 9, 10. So-construed, metacognition would encompass every type of cognitive control. We define metacognition more narrowly, as control processes that make use of one or more metacognitive representations, that is, representations of a property of a cognitive process (Box 1, Box 2).

Box 1 **Metacognition in humans**

Metacognition can be assessed with a range of different paradigms. Perceived (in)correctness of a first-order decision is often measured retrospectively through explicit second-order confidence or error detection judgements 58, 59. Moreover, post-decision wagering requires participants to place a wager on a just made decision, assuming higher bets will follow from higher confidence [60]. Another measure often used in reward paradigms is opt-out or uncertainty response, which allow the participant to skip a response, obtaining a smaller but guaranteed reward [61]. All these judgements are diagnostic of task performance, meaning higher confidence on correct compared with error trials. This leads to the question as to what metacognitive information is being represented by system 1 and system 2 metacognition. Studies have identified a range of candidates, which can be divided into two classes: directly accessed and inferred 39, 62.

Direct access models assume that metacognition is based on the same information as the decision itself or some property of the decision. There are cases of direct access system 1 metacognitive representations that are based on exactly the same information as the decision. For example, Gigerenzer and colleagues suggested that both decision making and confidence are based on the validity of an activated cue [63]. A similar assumption is made by type 2 signal detection theory [64]; in this class of direct access models, confidence corresponds to the quantity of decision evidence accumulated for one response option. Other properties of the decision evidence have been suggested to play a role, such as its quality. Both Peirce's model of confidence [65] and Kiani and Shadlen [4] suggest a combination of both 58, 59. The idea that external variability affects confidence has also been suggested [59], meaning that more variable stimuli lead to lower levels of confidence. Others have suggested cognitive conflict as a basis for metacognition 13, 17, which is based on the idea of competing response tendencies. The evidence in favour of the unchosen choice option also plays a role in mismatch models, which assume that metacognition is based on an internal matching process of the intended action with the actually performed one 66, 67, as well as prediction errors [46]. Similarly, the balance-of-evidence hypothesis is another example of direct access models, according to which, confidence is a function of the evidence for the chosen and the unchosen option [68].

In contrast to direct access models, inferential models assume that metacognition is based on information external to the first-order decision-making process. For example, an individual may learn that information from conflicting or variable sources of evidence leads to slower responses [69], and thus infer her confidence from response times, either approximated internally 70, 71 or through the observation of one's own or another individual's response movement. Several other inferential models, rooted in the metamemory literature, assume that metacognition is based on ease of processing, that is how accessible a representation is [72], how fluently it can be processed [73], or how familiar it is [74]. Another inferential hypothesis is the self-consistency model (SCM 39, 75), based on the consensuality principle, which assumes participants have implicit knowledge of what response others would give and approximate their confidence accordingly.

Box 2 **Metacognition in non-human animals**

There is compelling evidence that non-human animals are more likely to seek additional information 76, 77, to opt out of making decisions 4, 5, 78, 79, 80, and to make lower post-decision wagers 67, 81 under conditions in which a human observer would describe them as uncertain; for example, when the animal is required to make a difficult rather than an easy visual discrimination, or to remember an event over a long rather than a short interval. Some recent studies of monkeys [67], rats 5, 78, and pigeons [79] have also indicated, using transfer tests and single neuron recording, that this type of metacognitive behaviour can be regulated by internal rather than external cues; for example, that it covaries more precisely with neural signals from the orbitofrontal cortex or the supplementary eye fields than with external stimulus values.

These data suggest that animals from a wide range of species are capable of system 1 metacognition. As we have characterised it, system 1 metacognition is undemanding (see subsection ‘Only some forms of cognitive control involve metacognition’ above). It could be mediated by simple reinforcement learning (cf. 7, 82). However, the current data do not show that animals are capable of system 2 metacognition: that they are able to infer or to learn, using system 2, the metacognitive significance of system 1 cues. Even in the most compelling experiments (e.g., 60, 78), it is possible that a ‘high confidence’ internal system 1 signal, X, automatically triggered reward-seeking behaviour – and thereby reduced the probability of information-seeking, opt-out, and low post-decision wagers – without the animal having to use a system 2 process to learn that, in the presence of X, reward-seeking behaviour tends to be successful.

For example, a perceptual process may use the mean firing rate of a population of neurons in visual cortex to represent a perceived property such as the length of a line. Crucially, the variance of the firing rate across the population indicates the extent to which the neurons ‘agree’ about the line length and thus carries information about the reliability of the perceptual representation (metacognitive information) [11]. We propose that, if the latter type of information is used to control the relative influence of the perceptual representation on cognition [12], then it counts as a metacognitive representation, and the use of this representation for cognitive control should be considered to be an instance of metacognition. As well as choosing which sensorimotor dispositions are allowed to drive behaviour, metacognition thus conceived can be involved in distributing resources between rival processes [13], in emotion regulation [14], in guiding memory retrieval [9], allocating study time [9], and so on.

### Only some forms of cognitive control involve metacognition

Although all metacognition is a form of cognitive control, only some forms of cognitive control are metacognitive. Cognitive control need not make use of metacognitive representations and can be guided instead by object level representations, for example, of value or of abstract goals 15, 16. By contrast, other control processes do make use of metacognitive representations, for example, they represent that mutually inconsistent actions have been activated, which prompts the use of cognitive control processes in response selection 10, 17.

Thus, our first conclusion is that, if metacognition is defined carefully, metacognition does not encompass every type of cognitive control. What, then, is distinctive about metacognition, beyond its defining feature – the use of metacognitive representations? One possibility is that metacognition is only found in the cognitive control processes carried out by higher executive systems. In considering this possibility and suggesting an alternative, we make use of the well-known distinction between system 1 and system 2 [3]. However, our proposal does not depend on the details of dual systems theory, and it is consistent with other theories of higher-level executive function 18, 19, 20. The dual-systems model draws on evidence that there is a distinctive form of cognitive processing, type 2 processing or system 2, with respect to which various functional features tend to cluster together, underpinned by their reliance on a capacity-limited generalised working memory system 2, 3, 21. System 2 handles problems serially, takes time to operate and is affected by general working memory load. Its effectiveness correlates with individual differences in measures of general intelligence. By contrast, system 1 processes can act quickly, with many operating autonomously in parallel, and are little affected by general working memory load. System 2 representations are characteristically explicit, that is, conscious, whereas system 1 representations need not be.

Are metacognitive processes simply those control processes that are carried out by system 2? Unfortunately, things do not fall out so neatly. Much cognitive control takes place outside system 2 22, 23. For example, experimental subjects can inhibit responses to stimuli of which they remain unaware due to visual masking [22] and slow down after errors they do not know they have made [24]. These system 1 types of control can make use of metacognitive representations such as decision uncertainty 25, 26, for example, as evident in a neuropsychological patient whose behaviour shows adaptation to the prevailing difficulty of the task even though she has no conscious experience of mental effort [25]. Non-human animals are similarly capable of using metacognitive information [27] (Box 2). That, however, leaves us with a pressing puzzle. If system 1 metacognition is so pervasive, in humans and other animals, what role is there for system 2 metacognition?

## A hypothesis about the distinctive function of system 2 metacognition

Our answer to the puzzle – what is the role of system 2 metacognition? – will be that it comes into its own when the action-driving systems to be controlled are found in two different agents: that is, in cases of ‘supra-personal cognitive control’.

**Figure 1:**
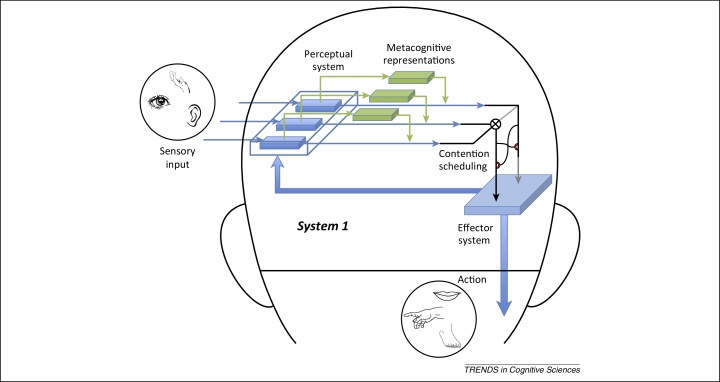
System 1 metacognition with a single agent. The control of cognitive processes in an individual's system 1 occurs automatically and at a sub-personal level [3]. Contention scheduling, a term introduced by Norman and Shallice [18], refers to a mechanism for resolving competition between processes that overlap in their effector system requirements. Metacognitive representations reflect the properties of the functioning of these cognitive processes. System 1 uses metacognitive representations to improve control. An example of such representations would be the reliability of sensory signals. Cognitive processes estimated to be more reliable can be given greater weight [12].

Within a single agent, domain-general processes of learning, which are part of system 1, can quickly establish – through experience – the best way to prioritise the inputs to and outputs from competing sensorimotor systems (Figure 1). Such processes can use system 1 metacognitive information – for example, variance of a distribution, time to completion of a process, activation of incompatible response tendencies (Box 1) – to improve those trade-offs, just as they can learn to use any other type of relevant cue. The result will be a form of control that is implemented within system 1 processing and relies on metacognitive representations – that is, a system 1 form of metacognition.

**Figure 2:**
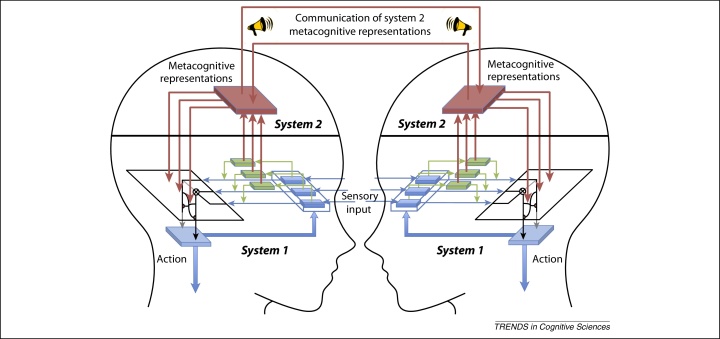
System 2 metacognition for cognitive control across two agents. System 2 metacognitive representations are derived from information in system 1, but they are in a form available for verbal report. For example, the reliability of a sensory signal can be reported in terms of confidence. When agents are cooperating, these reports can be used to optimise control by, for example, giving more weight to the more confident observer [32]. Via system 2, verbal reports can also have long-term effects on the functioning of system 1 [57].

When sensorimotor systems have to be coordinated between two or more interacting agents it is no longer possible for learning automatically to make use of all and any metacognitive information located anywhere within the different agents: my system 1 learning processes have direct access to the metacognitive information in my head, but not to the metacognitive information in your head, and vice versa. Some forms of coordinated action do not depend on metacognitive representations, for example, bodily movements can be synchronised relying only on object level information (e.g., about the location and trajectory of limbs) 28, 29. But inter-agent control will typically be more effective when it can use metacognitive representations, if relevant metacognitive representations within system 1 processes in each agent are selected for broadcast to the other agent, so that decisions about which sensorimotor processes to deploy can be taken in a space of shared metacognitive information. This, we suggest, is the distinctive role of system 2 metacognition: to select metacognitive information for broadcast, in the service of controlling the sensorimotor systems of two or more agents involved in a shared task – that is, for supra-personal cognitive control (Figure 2).

Ours is different from the hypothesis that metacognition is for mentalising –that is, perceiving or inferring other people's mental states 30, 31. The first difference is that the representations concern one's own cognitive processes in the first instance, rather than those of another. The second difference is that communicating metacognitive representations plays a central role. To caricature: the mentalising story says that metacognition is there to allow agent A to infer that agent B has seen a rabbit; according to us, system 2 metacognition is there to allow agent A to communicate that his visuomotor fix on the rabbit is pretty reliable at present.

## How system 2 metacognition works

### System 2 metacognition plays a central role in group decision making

According to our hypothesis, system 2 metacognition should play a central role in group decision making. Indeed we find that joint perceptual decision making is significantly more effective when participants communicate metacognitive representations about the stimuli 32, 33. When deciding whether to trust witness testimony, (mock) jurors make considerable use of a witness's confidence and other metacognitive representations (e.g., calibration of confidence and accuracy) [34]. People also communicate metacognitive representations when they are synchronically coordinating complex actions (e.g., communications about confidence used in team sports) 35, 36. System 2 metacognition can also be used diachronically, for example, making it possible for people to discuss how metacognitive representations should be deployed, affecting their own cognitive control [37]. Control strategies based on metacognitive representations, for example, what to do when memory fails, can be the subject of explicit instruction. For example, a person can learn socially that, following failure to produce items in a verbal fluency task to name animals, a good strategy is to consider subcategories of animals (farm animals, pets, etc.) [38].

### Three types of work done by system 2 metacognition

We can distinguish three different types of work done by system 2 metacognition. First (W1), it makes metacognitive representations available for verbal report and hence for communication. Second (W2), system 2 metacognition works out the significance of metacognitive representations that have been broadcast or selected for communication, that is, what the individual or group should do (e.g., try harder, start again, give low weighting to that person's opinion) when a metacognitive representation has a certain range of values (e.g., indicates disfluency or lack of confidence). Although system 1 processes already contain some metacognitive representations which system 2 metacognition can select to make available (‘direct access’ [39]), a third type of work done by system 2 metacognition (W3) is to construct or infer metacognitive representations from multiple sources of (sometimes weak) metacognitive information (Box 1, Figure 2).

In addition, we can, as mentioned above, distinguish between synchronic and diachronic supra-personal cognitive control. In cases of synchronic coordinated agency, metacognitive representations are used to improve the performance of two or more people when working on the same task at the same time. Metacognitive representations can also be used diachronically in teaching other agents how to think and act in the future, to improve subsequent performance in coordinated action [40], and possibly also in solitary tasks [41]. The interpretation of metacognitive representations, and what to do about them, can be altered through discussion with others, enabling the generation of cultural consensus and regulation concerning what the cues mean and why some instructions are more appropriate than others. For example, using system 2 metacognition, groups develop and pass on theories about system 1 processes (about how to think) [37]. Learnt reliance on shared metacognitive representations can be effective even though people can be very unreliable in reporting some of the object level information used by system 1 processes. For example, people are wildly incorrect about the object level information used by the system 1 processes involved in catching a ball, but can still share metacognitive information to facilitate joint performance 35, 42.

When is system 2 metacognition direct and when is it inferred (W3)? We suppose that direct access will be used where the information is available, for example, confidence in perceptual decision making; and inferred otherwise, for example, in predictive ease of learning judgements 9, 43 (Box 1). If direct, there should be a reasonably tight correlation between the subject's reports (e.g., of accuracy) and objective measures 44, 45. If system 2 has to infer the metacognitive significance of system 1 cues there is more room for error.

### Examples and limitations of system 2 metacognition

One central source of metacognitive representations is the prediction errors generated and used by many system 1 processes to guide learning [46]. When relied on to guide learning and behaviour prediction errors can represent the reliability of another of the system's representations (e.g., of the value of an option) and lead that representation to be revised for the future [47]. Large prediction errors may produce feelings of perceptual disfluency [48] or action selection disfluency [49]. System 2 can learn the significance of these feelings and use them for cognitive control.

System 2 has limited processing capacity, thus it can only make use of a limited number of metacognitive representations. That number should decrease as other demands on general working memory increase. And indeed there is some evidence that under cognitive load subjects switch from relying on several raw cues to depending on a single summary [50]. An example of such a summary is the z-score (derived from the mean and the standard deviation of the internal perceptual representation) communicated by subjects in social psychophysics experiments [32], although it is not known whether subjects could have communicated the raw cues independently. In some cases, the raw cues on which summaries are based may no longer be available. However, this mandatory fusion occurs relatively late in development (∼12 years [51]), suggesting that adults could learn to unpack the summary if necessary.

## The function of system 2 metacognition

### The functional claim about system 2 metacognition comes in various strengths

We have suggested that system 2 metacognition is for supra-personal cognitive control. The functional claim – what system 2 metacognition is ‘for’ – comes in several strengths. A modest claim is that system 2 metacognition is functionally involved in cases of inter-agent cognitive control; in situations where two or more people successfully coordinate their actions to achieve an outcome that depends on sensorimotor processes found in both. A bolder hypothesis is that this is the evolutionary purpose of system 2 metacognition: that the ability to represent metacognitive information in system 2 evolved to allow people to engage in more sophisticated cooperative projects and coordination tasks.

### The hypothesis that system 2 metacognition evolved for supra-personal cognitive control

There is not scope here to properly assess the evolutionary claim, but we will note an empirical consequence. Humans are a distinctively cooperative mammal, engaging in coordinated behaviour to an extent that is markedly different from even our closest primate cousins 52, 53. If the ability to use system 2 to select, construct, and broadcast metacognitive representations arose in humans in response to selection pressure for increasingly complex forms of coordinated action, then in non-human animals we would not expect to find a system 2 that processes metacognitive representations (Box 2). One possibility is that non-human animals do not have a system 2 at all. Alternatively, non-human animals may have system 2 functions, such as maintenance in working memory and selective attention 54, 55, that do not involve the use of metacognitive information. In this case, what evolved in the hominin line was the capacity to take metacognitive representations in system 1 and turn them into metacognitive representations for system 2.

According to this evolutionary hypothesis, supra-personal coordination was the origin of system 2 metacognition. Using conscious metacognitive representations to control one's own individual behaviour is probably the most obvious manifestation of system 2 metacognition in everyday life. But on our account, using metacognitive representations about one's own cognitive processes for intra-personal cognitive control came second, and arose as a side effect of the selection of system 2 metacognition for inter-personally coordinated action. The picture is of cooperation becoming a central feature of the human way of life, in the form of fluidly coordinated joint action and diachronically organised cooperative projects, both controlled by a supra-personal system of cognitive control. This system directs resources and activity between a variety of different sensorimotor processes that are distributed across agents. It relies on selected metacognitive representations which, because they are shared among the agents involved, enable more efficient and complex forms of coordinated action. The selection and sharing occurs not only synchronically, while a particular episode of coordinated action is in progress, but also diachronically, as system 2 metacognition allows experts to teach novices how to use metacognitive representations in future episodes of coordinated and solitary tasks.

The selection processes favouring the emergence of system 2 metacognition could have been genetic and/or cultural. To the extent that the evolutionary processes were genetic, rather than cultural, one would expect: (i) little cross-cultural variation in the extent and content of system 2 metacognition, especially when people are tested in coordinated action tasks and when adults are teaching children; and (ii) that metacognitive competence would emerge early and independently of instruction. If these predictions are false [56], it would suggest a role for cultural selection in the evolution of system 2 metacognition (see section on ‘Empirical predictions’ below).

### An (even) more radical evolutionary hypothesis about system 2 metacognition

The evolutionary claim about the function of system 2 metacognition in turn comes in various strengths. So far, we have suggested that it was the ability to represent metacognitive information in system 2 that was selected. This allows that early hominins may already have had system 2 in place, and perhaps also natural language (which may or may not be separable from the presence of system 2). However, it is worth noting that a much more radical evolutionary claim could also be entertained: that system 2 itself evolved to perform the function of selecting, constructing, and broadcasting metacognitive representations in the service of supra-personal cognitive control. A yet further evolutionary question on which we do not venture to speculate is the relation of these various evolutionary steps to the emergence of consciousness.

## Empirical predictions

We suggest several empirical predictions against which our hypothesis can be tested. The first concerns linguistic communication. It follows from our functional hypothesis that some forms of joint action are significantly aided by communication of metacognitive representations selected by system 2 metacognition. Thus, in a novel task requiring two or more agents to act jointly in pursuit of some goal (i.e., not one where an action plan has been automatised in system 1 processes in both agents), blocking the linguistic communication of metacognitive information should have a selective detrimental effect on performance, in a way that blocking the linguistic communication of object level information about the task does not.

Similarly, in such a novel coordination task, cognitive load should selectively impair performance, and should do so via an effect on the (accuracy or range) of communicated metacognitive information. By contrast, where automatic/autonomous/implicit processes rely on metacognitive information in directing behaviour or resources between sensorimotor processes, increased cognitive load should cause relatively little impairment.

A third prediction concerns individual differences. Performance in the types of novel coordination tasks just described should correlate with individual differences in measures of general intelligence.

Our final prediction concerns non-human animals. If, contrary to our hypothesis, non-human animals have system 2 metacognition, they should be able to learn that reward-seeking behaviour is successful after making decisions that are unlikely to be correct (low confidence) and unsuccessful after making decisions that are likely to be correct (high confidence). This could be tested by, for example, using a reverse transfer test after training in a wagering task.

## Concluding remarks

As yet the evidence for our hypothesis is limited and there are many outstanding questions (Box 3). The two key components, for which there is some preliminary evidence, are: (i) that system 2 metacognition is very malleable and readily influenced by instructions and beliefs 56, 57; and (ii) that exchange of system 2 metacognitive representations can create advantages for the group [32]. If tests of our empirical predictions provide further support, it would suggest that metacognition is a specific form of cognitive control and is not unique to system 2. Metacognitive information such as decision uncertainty is used to modulate ongoing thought and behaviour in the absence of awareness. These implicit metacognitive representations enable the many different processes that make up system 1 to work together in an optimal manner. However, when metacognitive representations are explicit in system 2 they can be readily used to enable several people to work together in an optimal manner, for example, by sharing information about decision confidence. We suggest that explicit metacognitive representations exist for this reason. This role is possible, first because the representations are in verbal form suitable for sharing, and second because they can modify the functioning of system 1. On the basis of metacognitive signals emerging from system 1, accounts are developed in system 2 designed to explain and control the functioning of system 1. These accounts can be sketchy and inaccurate, like any narrative, but are constantly updated and sometimes improved through discussion with others. This process provides a mechanism for modification of behaviour through instructions and cultural expectations. These are powerful forces that alter behaviour of whole groups of people and provide a mechanism for the emergence of cumulative culture.

Box 3 **Outstanding questions**

• Is there a specific format for system 2 metacognitive representations designed to enable a common supra-personal metric (e.g., confidence as signal mean*precision)?
• Can cognitive control be improved through inter-personal exchange of system 2 metacognitive representations?
• Can the interpretation of system 2 metacognitive representations be changed/corrected through discussion (e.g., learning that perceptual fluency indicates familiarity)?
• What are the distinctive features of group decision making in humans in comparison to eusocial animals such as ants and bees?
• Are there cultural differences in the use of system 2 metacognitive representations?

## Glossary

Cognitive control: cognitive mechanisms responsible for guiding thought and behaviour in accordance with current goals and intentions.
Conscious: here we use the term in the sense of access consciousness [1]. A representation is access conscious just in case it can be used, without further processing, for verbal report, inferential reasoning, storage in episodic and semantic memory, and by other ‘consuming’ systems.
Metacognition: use of metacognitive representations (often, but not exclusively, for purposes of cognitive control).
Metacognitive information: information about a property of a cognitive process, for example, the variance in the firing rate of a population of neurons in visual cortex. Information is just a matter of correlation, strong or weak, and need not be used or represented.
Metacognitive representation: a representation of a property of a cognitive process, for example, the reliability of a perceptual representation. Metacognitive information that is represented and used for cognitive control is thereby a metacognitive representation, for example, the variance in the firing rate of a population of neurons in visual cortex can form the basis of a representation of the probability that the perceptual representation is correct (cf. object level representation).
Object level representation: a representation that is not at the meta-level, that is, that does not concern cognitive processes as such. Examples include representations of the nature, location, or value of a stimulus. (Similarly, correlates of such properties, whether represented or not, carry ‘object level information’.)
System 1 (type 1 cognitive processes): a label for a number of cognitive systems that operate autonomously and do not require working memory. A system 1 process is typically fast, automatic, associative, effortless, and non-conscious. System 1 processes tend not to cause or suffer much interference when combined with one another or the performance of other tasks [2,3].
System 2: a label for a cognitive system that relies on working memory and is typically slower, serial, rule-based, more effortful, and conscious. Because of its limited capacity, processes relying on system 2 tend to disrupt each other [2,3].
